# Dairy Chain Safety in the Context of Antibiotic Residues—Current Status of Confirmatory Liquid Chromatography Methods: A Review

**DOI:** 10.3390/antibiotics13111038

**Published:** 2024-11-03

**Authors:** Sandra Dluhošová, Klára Bartáková, Lenka Vorlová, Pavlína Navrátilová, Oto Hanuš, Eva Samková

**Affiliations:** 1 Department of Animal Origin Food & Gastronomic Sciences, Faculty of Veterinary Hygiene and Ecology, University of Veterinary Sciences Brno, 61242 Brno, Czech Republic; dluhosovas@vfu.cz (S.D.); navratilovap@vfu.cz (P.N.); 2 Dairy Research Institute Ltd., 16000 Prague, Czech Republic; hanus.oto@seznam.cz; 3 Faculty of Agriculture and Technology, University of South Bohemia in České Budějovice, 37005 České Budějovice, Czech Republic; samkova@fzt.jcu.cz

**Keywords:** milk, HPLC, beta-lactams, tetracyclines, sulfonamides, (fluoro)quinolones, aminoglycosides, polypeptide antibiotics, legislation

## Abstract

With ever-developing analytical capabilities, the protection of the dairy chain from contamination by residues of veterinary drugs is improving. Legislative requirements are an inherent part of this process. Of antibiotics in dairy farming, representatives of the beta-lactams, tetracyclines, sulfonamides, (fluoro)quinolones, aminoglycosides, or polypeptide antibiotics are the most widely used. Due to the typically low levels of antibiotic residues in milk, mass spectrometry is the most commonly used detection technique. However, the interference of the sample matrix is one of its main limiting factors, and therefore, it needs to be eliminated. In the first step, the lipid fraction is removed and proteins are precipitated, followed by solid-phase or liquid–liquid extraction. The current trends include the reduction in the consumption of organic solvents (to reduce occupational hazards and burden to the environment) and automation, eliminating the influence of human error and optimizing the workflow. These trends lead to the development of new microextraction and automated techniques as well as the use of new sorbents and/or (green) solvents of natural origin. To capture the latest developments in the field and the relatively recent aforementioned trends, this review focuses on papers investigating antimicrobial residues in milk that were published between 2015 and 2024, providing an overview of emerging techniques applicable to antibiotic residue detection in milk samples.

## 1. Introduction

Ensuring food safety, along with promoting and restoring public health, has grown in importance throughout the food chain. Antibiotics are a large group of antimicrobial agents that have become irreplaceable in human as well as veterinary medicine since the discovery of penicillin by Alexander Fleming in 1928 [1]. They are used extensively for both preventive and therapeutic reasons. Besides their antimicrobial use, some of these substances were also previously used as growth promoters, supporting higher nutrient conversion and muscle mass yield. Historically, poultry and monogastrics (especially pigs) have been most affected by such use of antimicrobials. Due to the negative impacts of such practices on human and animal health, feeding antibiotics to livestock to improve their growth was banned in the European Union (EU) in 2006 [2,3,4,5,6].

Although the awareness of sustainability in food production has been increasing over the last decades, there is still pressure on maintaining high production levels of food animals (i.e., animals primarily bred and raised for production of human food) to supply the market with commodities such as meat and milk (or eggs and honey). As the use of antibiotics in farming is both traditional and effective, the risks of residues of these substances in raw foodstuffs persist. The presence of residues of antimicrobials in food leads to legitimate public health concerns associated with the emergence of resistant bacterial strains and the occurrence of more or less serious side effects of the consumption of foods contaminated with antibiotic residues (ARs), such as hypersensitivity, allergic reactions, fever, hemolysis, tooth discoloration, disruption of the gut microbiome, nephrotoxicity, hepatotoxicity, neurotoxicity, ototoxicity, cytotoxicity, attenuation of bone marrow formation, or carcinogenicity [5,7,8,9,10,11,12,13,14,15,16,17,18,19,20]. More (and more detailed) information on the influence of ARs in milk can be found, for example, in the review by Mathew et al. [21].

With the increasing knowledge in the field of residues of foreign agents (physical, chemical, and biological hazards) in the environment and/or food chain [5], the global antibiotic policy is to progressively reduce the consumption of antimicrobials in both animal and human medicine [22,23,24,25]. The so-called anatomical–therapeutic–chemical classification of drugs according to the World Health Organization (WHO) is helpful in navigating the groups of drugs used [26].

In veterinary medicine, antimicrobials are often used in milked ruminants for the management of mastitis, which can lead to the release of pathogenic or non-pathogenic bacteria as well as residues of inhibitory substances into the primary foodstuff (e.g., milk) intended for human consumption. In the case of milk, this may also affect, besides the consumers’ health, its processing into dairy products. The presence of antibiotic residues can affect the fermentation capacity of bacteria used in the manufacturing of fermented dairy products such as yogurt, cheese, or butter [5,27]. This may lead to secondary economic losses in the food industry, for example, through creating barriers to international trade in dairy products. When importing milk or dairy products into another country, the legislative parameters (maximum residue limits, MRLs) of the importing country must be followed. The MRLs set in the Codex Alimentarius [28] are based on the results of studies by the WHO and the Food and Agriculture Organization of the United Nations (FAO). Individual countries either adopt them into their legislation or adjust the limits based on their own scientific studies.

The variation in MRLs for different antibiotics in different countries or unions, as well as the differences in the sensitivity of tests used in individual countries for the determination of residues of inhibitory substances (RISs, mainly antibiotics), poses challenges to the unhindered international trade in milk. The different practices in dairy farming among countries can, for example, lead to different levels of contamination of powdered milk, which can be easily transported internationally, or to issues related to international dairy product trade between neighboring countries with different MRLs.

The prevalence of ARs in raw milk differs even among countries with identical MRLs. In EU countries, however, the prevalence is quite similar. For example, the AR prevalence in milk samples delivered to dairies in the Czech Republic ranged in recent years approx. from 0.01 to 0.24% [29], and similar results can be seen also in other EU countries. In Bavaria, for example, a decrease of 50% in the prevalence of ARs in raw milk from 0.05 to 0.025% between 2006 and 2016 has been reported [30]. On the other hand, a recent Colombian study detected beta-lactam residues in 5.9% of raw milk and 29.4% of commercial milk samples [31]. Such high residue levels can, of course, pose a barrier to international trade.

Methods applied to the determination of residues of undesirable substances in food and other matrices are crucial for detecting non-compliant, unacceptable, or deceptive practices in the production, distribution, and sale of food. Efforts are continuously ongoing to develop the best analytical methods meeting the criteria of speed, simplicity, economy (time, money, personnel, chemical reagents), and efficiency while achieving accurate and understandable results. The development of such methods is an extremely dynamic process, which must take into account the nature of the food matrix to be analyzed, the chemical reactions taking place within it, the interacting substances present, the actual sample preparation, and the final analysis of the sample. Sample preparation should ideally support the extraction of most of the analytes of interest (belonging to different groups of chemicals) from the matrix using the same operational procedure. Effective detection methods for routine laboratory testing should meet the requirements for multi-residue analysis in the shortest possible time. The task of developing methods covering a wide spectrum of analytes is, therefore, highly challenging. In this review, we aimed to capture the developments in this field between 2015 and 2024 to give a comprehensive picture of the state-of-the-art methods that can be used for this purpose—methods for the analysis of individual antimicrobials as well as multi-residual analyses comprising tens of analytes are included.

## 2. Legislation

Generally, the guidelines for the evaluation of risks and the surveillance of veterinary drug residues in food products sourced from animals are comparable globally. The Joint Food and Agriculture Organization/World Health Organization Committee of Experts on Food Additives (JECFA) is an example of an independent risk assessment body. In the U.S., this area is governed by the Food and Drug Administration (FDA); in the EU, this role is played by the European Medicines Agency (EMA) [32]. As examples of legislative documents governing this field and establishing MRLs in milk, we can name the EU Regulation 37/2010 [33]; in the United States, it is CFR—Title 21, PART 556 [34]. The use of antibiotic growth promoters (EU: Regulation 1831/2003 [2]; USA: Guidance for industry #213 [35]) or drug residues in food products imported from third countries (EU: Commission Regulation 2019/1871 [36]; USA: CFR—Title 21; PART 510 [37]) are dealt with in separate documents [38,39].

Most countries may have their own systems for assessing the presence of antibiotic residues in food [40,41]. Still, setting higher MRLs in milk does not only pose a greater risk to consumers’ health but also leads to issues in dairy plants (inhibition of starting cultures, negative impact on curdling and titratable acidity). Other countries, such as India, even lack any MRLs whatsoever; even though some guidelines limiting the use of antibiotics exist there, they have never been implemented into laws or regulations [21].

The legislation requirements and the need to comply with them directly affect the economy of dairy farms and processors. The AR levels in milk are among the key parameters of the procured milk quality. If the conditions are not met, milk must be disposed of as a Category II animal by-product. This inevitably entails both economic losses and a burden on the environment and, in effect, the food chain. For this reason, it is essential that supervisory authorities (and other bodies investigating the quality of milk) have methods and analytical techniques capable of determining the presence of ARs at the level of the limits set by legislation.

In this section, we will mention the EU legislation and also previously existing regulations that have been re-evaluated over time and replaced by newer regulations that are in line with new research findings and current knowledge.

In 1990, the Council Regulation 2377/90 [42] was passed, laying down procedures for the establishment of maximum residue limits for veterinary medicinal products in foodstuffs of animal origin. The Annexes to that regulation included lists classifying pharmacologically active substances into (i) those for which MRLs can be set; (ii) those not subject to such limits; and (iii) pharmaceuticals for which MRLs cannot be set. That document was later replaced with Regulation 470/2009 [43], which was supplemented with lists of prohibited pharmacologically active substances (*Aristolochia* spp. and preparations thereof, chloramphenicol, chloroform, chlorpromazine, colchicine, dapsone, dimetridazole, metronidazole, nitrofurans including furazolidone, ronidazole) and allowed substances (including their MRLs) published within Regulation 37/2010 [33]. For some of the authorized substances, this regulation provides more detailed specifications for use in milk ruminants, such as “Not to be used in animals whose milk is intended for human consumption”. This concerns apramycin and paromomycin (aminoglycosides), difloxacin (fluoroquinolones), doxycycline (tetracyclines), florfenicol (amphenicol), gamithromycin, tildipyrosine and tulathromycin (macrolides), oxolinic acid (quinolones), and lasalocid (polyether ionophore). Table 1 shows the overview of MRLs for individual antibiotics in milk based on Regulation 37/2010 [33]. For comparison, it also shows the MRLs set by the Codex Alimentarius, aiming to protect consumer health and ensure fair practices in the food trade. The Codex Alimentarius MRLs are used by some countries to regulate the presence of antibiotic residues [28].

The use of substances with hormonal or thyrostatic action and beta-sympathomimetics in animal husbandry remains prohibited (Council Directive 96/22/EC [44]). For certain other substances (including antimicrobials) and their residues, the Regulation 2017/625 [45] fully replaced obligations implied by the older Directive 96/23/EC [46]. This canceled directive classified substances into Group A (substances with anabolic effects and unauthorized substances) and Group B (veterinary drugs and contaminants). In addition, it sets the framework for monitoring individual foods, sampling requirements, and reference laboratories. The Directive 2017/625 [45] details, besides the rules for sampling and inspection by the supervisory authorities, the criteria that need to be met by analytical methods used by the authorities for the analyses of inspection samples.

More specific requirements on the performance of analytical methods and the interpretation of results, including the definition of validation parameters (such as precision or recovery) and detection methods applicable after the chromatographic separation of analytes used in official laboratories, were the subject of Commission Decision 2002/657/EC [47], which was replaced by Commission Implementing Regulation 2021/808 [48]. Certain requirements of the Decision 2002/657/EC [47] remain in force until 2026. Regulation 2021/808 [48] also took over the content of the Commission Decision 2003/181/EC [49] on the minimum required performance levels (MRPLs) for substances for which MRLs cannot be set. The MRPLs apply, e.g., to chloramphenicol content in meat, eggs, milk, aquaculture products, and honey.

Commission Decision 2005/34/EC [50] laying down harmonized standards for the testing of certain residues in products of animal origin imported from third countries was replaced with Commission Regulation 2019/1871 [36]. The regulation sets out the reference points for action for unauthorized pharmaceuticals in foods of animal origin. These limits include chloramphenicol (0.15 µg/kg), malachite green (sum of malachite and leucomalachite green 0.5 µg/kg), and nitrofurans and their metabolites (0.5 µg/kg for each metabolite).

In 2004, Regulation No. 726 [51] detailing EU procedures for the authorization and supervision of medicinal products for human and veterinary use was adopted, establishing also the European Medicines Agency. In 2019, this regulation was updated (Regulation 2019/5) [52].

Regulation 2019/6 [53] replaced the Directive 2001/82/EC [54]. The directive established the code of practice for veterinary medicinal products, requirements, and procedures for the analytical testing, safety testing, and pre-clinical as well as clinical evaluation of veterinary medicinal products. Regulation (EU) 2019/6 [53] also addresses the issues of the availability and safety of veterinary medicinal products, antibiotic resistance in association with the responsible use of antimicrobials in food animals, pharmacovigilance rules, and effectiveness of the market for veterinary medicines.

## 3. Antibiotics in Food Animals

Antimicrobials most commonly used in dairy cattle husbandry, i.e., the most important group from the perspective of the need for monitoring and residue capture, include beta-lactams [3,9,13,20,55,56], tetracyclines and their epimers [5,7,8,14,18,20,55,56], sulfonamides and their acetylated metabolites [7,14,16,17,20,56,57], (fluoro)quinolones [3,5,9,11,14,16,20,55,56], aminoglycosides [6,58,59], macrolides [19,20,56], polypeptide antibiotics [4,59], and amphenicols [12,55]. Each group of antimicrobials comprises a variety of representatives, of which those most commonly observed in milk are listed in Table 1. For this reason, the attention of researchers focuses either just on a single or a few selected representatives within each group or on multi-residue analyses capable of capturing multiple groups of antibiotics and other veterinary drugs within a single run (tens to hundreds of analytes in a single analysis) [10,20,60].

Thanks to their broad-spectrum effect on Gram-positive and Gram-negative bacteria, chlamydia, rickettsiae, mycoplasmas, or protozoan parasites, the above-mentioned groups of antimicrobials are extensively used in both human and veterinary medicine. Their use in both local and systemic infections is supported by their easy availability, high efficacy, low cost, and the possibility of various forms of administration. In milked cattle, their use in the treatment of mastitis is important [4,6,7,9,12,13,18,19,57,59].

The use of antibiotics in animal husbandry brings significant risks to human health, (such as the development of antimicrobial resistance). Based on the increasing risk, several strategies for classifying antibiotics have been proposed. The World Health Organization has proposed the categorization of antibiotics from the perspective of human medicine. The sixth revision issued by the WHO focuses on antibiotics important for human health while taking into account the transfer of antimicrobial resistance genes from animals to humans [61]. Other categorizations of antibiotics have been proposed by the World Organization for Animal Health (WOAH, formerly OIE) and the European Medicines Agency (EMA), focusing on antibiotics used in animals [62,63]. The current, revised, categorization of antibiotics issued by the EMA takes into account two main criteria: the potential risk of veterinary antibiotics to promote the development of resistance with regard to public health and the importance of the respective antibiotics in veterinary medicine. The EMA classifies antibiotics into four categories, designated as A to D. All these categories are applicable to both food animals and companion animals:

**Category A (“Avoid”)** includes antibiotics that are currently not authorized in veterinary medicine in the European Union. These medicines may not be used in food-producing animals at all; under exceptional circumstances, however, they may be given to individual companion animals.

**Category B (“Restrict”)** refers to quinolones, third- and fourth-generation cephalosporins, and polymyxins. Antibiotics in this category are critically important in human medicine, and their use in animals should be restricted to mitigate the risk to public health.

**Category C (“Caution”)** covers antibiotics for which alternatives in human medicine generally exist in the EU, but few alternatives are available in certain veterinary indications. These antibiotics should only be used when there are no antimicrobial substances in Category D that would be clinically effective.

**Category D (“Prudence”)** includes antibiotics that should be used as first-line treatment whenever possible. These antibiotics can be used in animals in a prudent manner. This means that unnecessary use and long treatment periods should be avoided, and group treatment should be restricted to situations where individual treatment is not feasible.

The antibiotics in Table 1 are color-coded according to the A–D categories [63].

**Table 1:** Values of the maximum residual limits (MRLs) for antibiotic residues in milk (based on Regulation 37/2010 [33] and Codex Alimentarius [28]). Color-coding of the antibiotics corresponds to the EMA categorization of the potential risk of resistance development (category B (“Restrict”) = red; category C (“Caution”) = orange; category D (“Prudence”) = green) [63].

Antibiotic Group	Characteristics of Antibiotic Groups [40]	Analyzed Substance		MRLs in Milk [µg/kg]	
				According to the EU Regulations [33]	According to the Codex Alimentarius [28]
Amphenicols	Broad-spectrum antibiotics with bacteriostatic effects.	Thiamphenicol		50	Not determined
Aminoglycosides	The most common aminoglycosides in food-producing animals are streptomycin, gentamicin, and neomycin, although other members of this family are less commonly used. Gentamicin is an aminoglycoside complex with the broadest range of activities among aminoglycosides, which is used in cattle to treat mastitis or respiratory diseases. Neomycin is a broad-spectrum antibiotic used to treat bacterial infections in cattle.	Dihydrostreptomycin		200	200
		Gentamicin		100	200
		Kanamycin		150	Not determined
		Neomycin		1500	1500
		Spectinomycin		200	200
		Streptomycin		200	200
Beta-lactams	The most common and widely used veterinary antibiotics frequently used in animals in feed for growth promotion and treatment of bacterial infections and mastitis. They are classified according to a common structural feature, the beta-lactam ring.	Penicillins	Amoxicillin	4	4
			Ampicillin	4	Not determined
			Benzylpenicillin	4	4
			Dicloxacillin	30	Not determined
			Cloxacillin	30	Not determined
			Oxacillin	30	Not determined
		Cephalosporins	Cefacetrile	125	Not determined
			Cefalexin	100	Not determined
			Cefapirin	60	Not determined
			Cefazolin	50	Not determined
			Cefquinome	20	Not determined
			Cefoperazone	50	Not determined
			Ceftiofur	100	100
Lincosamides	Macrolide-like antibiotics that act primarily bacteriostatically on Gram-positive bacteria, including penicillin-resistant strains. Furthermore, they are characterized by a significant effect on anaerobic microorganisms.	Lincomycin		150	150
		Pirlimycin		100	100
Macrolides	Very effective against Gram-positive bacteria, while many Gram-negative bacteria are resistant to macrolides because their outer membranes are impermeable to macrolides. Tylosin is widely used in veterinary medicine and bovine respiratory diseases.	Erythromycin		40	Not determined
		Spiramycin		200	200
		Tilmicosin		50	Not determined
		Tylosin		50	100
Polypeptides	Polypeptide antibiotics have bactericidal properties. Their spectrum mainly includes Gram-positive cocci and rods, especially some types of clostridia.	Bacitracin		100	Not determined
		Colistin		50	50
(Fluoro)Quinolones	Synthetic antibiotics that are very effective in animal husbandry. Fluoroquinolones are the second generation of quinolones, and their primary use is for gastrointestinal and respiratory infections. Enrofloxacin is the most commonly used fluoroquinolone in the European Union.	Sum of enrofloxacin and ciprofloxacin		100	Not determined
		Danofloxacin		30	Not determined
		Flumequine		50	Not determined
		Marbofloxacin		75	Not determined
Rifamycins	Only rifaximin from this group can be used to treat severe bacterial infections of the female genital tract.	Rifaximin		60	Not determined
Sulfonamides	Bacteriostatic antibiotics that inhibit the synthesis of nucleic acids, affect microorganisms, and kill a wide range of Gram-positive and Gram-negative bacteria. As a result, they lead to the treatment of many infections and increase the growth of animals in the livestock industry.	All substances belonging to the sulfonamide group		100	Not determined
		Sulfadimidine		Not determined	25
Tetracyclines	Tetracyclines have a bacteriostatic effect on a wide range of aerobic and anaerobic bacteria, both Gram-negative and Gram-positive. They are used for the prevention and treatment of diseases and as growth-promoting additives in animal feed at doses lower than the therapeutic dose.	Chlortetracycline		100	100
		Oxytetracycline		100	100
		Tetracycline		100	100
Trimethoprim	Trimethoprim acts chemotherapeutically in the treatment of primary bacterial infections and in the treatment of secondary infections during viral diseases.	Trimethoprim		50	Not determined

## 4. Quantification of Antibiotic Residues

The growth-inhibition-based screening test methods for the determination of the presence of antibiotic residues are not always reliable due to the possible false positive results (that can be caused by increased somatic cell counts as well as lysozyme and/or lactoferrin concentrations during infections, as well as fat and protein content in milk) [5].

For this reason, various types of liquid chromatography equipped with a variety of possible detectors are nowadays predominantly used as routine methods for the determination of antimicrobials in milk. According to the legislation [48], mass spectrometry detection (in which the substance is determined based on the mass of the molecule and its fragments) is the key confirmatory method. This method is highly reliable, sensitive as well as selective, and fast. However, as only trace amounts of ARs are present in milk, matrix interference can negatively affect both the accuracy and precision of the determination of such residues. To reduce the matrix effects, thorough sample preparation before analyte determination is crucial [57].

Due to the different chemical nature of different antibiotic groups (lipophilicity, hydrophilicity, acid–base characteristics, etc.), it is extremely difficult to develop a method for the determination of all potential residues in a sample. The parameters of the best-performing methods used in individual papers are shown in Table 2, which lists the type of detection used, the column parameters, and the mobile phase composition and characterizes the methods using selected validation parameters, namely the recovery (percentage of the actual concentration of the substance obtained during the analytical procedure), precision (closeness of agreement between independent test results obtained under specified (predetermined) conditions; the precision is usually expressed as the standard deviation of the test result), the limit of quantification (lowest concentration of analyte that can be reliably quantified), and the limit of detection (lowest concentration of analyte that can be reliably detected) [47].

### 4.1. Milk Sample Preparation for AR Determination

In most studies, the milk sample is first processed to maximize the efficiency of the subsequent extraction of antibiotic residues, which uses (mainly) solid-phase extraction (SPE) or liquid–liquid extraction (LLE). For clarity, this chapter is, therefore, divided into two parts—the first one describes the procedures used to prepare the milk for the extraction of the analyzed antibiotics, and the second part describes the extraction methods themselves. The cited works are arranged in ascending order of the year of publication (starting from 2015) to illustrate the gradual development in the field.

#### 4.1.1. Treatment of Milk Samples Prior to Antibiotic Residue Extraction

**Figure 1:**
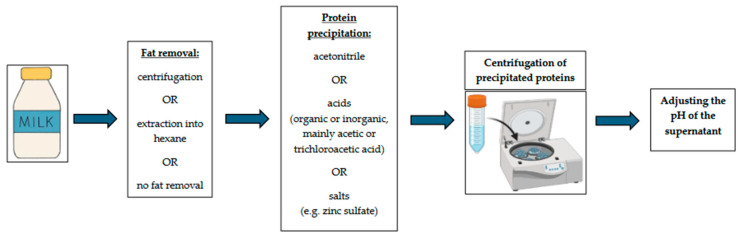
The schema of the workflow of milk sample pre-treatment before AR extraction (usual steps in cited studies).

The primary treatment of the milk sample aims at eliminating the interfering effect of sample matrices, represented in milk samples mainly by proteins and fat. The precipitation and subsequent removal of proteins from the milk is the most important step, as the imperfect elimination of proteins could cause contamination, blockage, and irreversible adsorption onto the stationary phase of the HPLC column or solid adsorbents. Solvents (in particular acetonitrile), or acids (organic or inorganic, mainly acetic or trichloroacetic acid), are commonly chosen for protein precipitation. The acids are often diluted in methanol or acetonitrile. The fat fraction can be removed through centrifugation or extraction into hexane before protein precipitation. Extraction processes are influenced by factors such as the solvent strength, target medium (organic or aqueous), sample pH, and the ability of the analyte to dissociate. In particular, an inappropriate pH may cause inefficient sorption and desorption on stationary phases. That is why the pH is often adjusted after protein precipitation. Some groups of antimicrobials need special treatment—for example, a chelating agent (especially Na_2_-EDTA [Disodium ethylenediaminetetraacetate] or Na_4_-EDTA [Tetrasodium ethylenediaminetetraacetate dihydrate]) needs to be applied in the case of tetracycline determination to disrupt the chelate complexes that tetracyclines typically form with calcium in milk, as such complexes cause a decrease in extraction yield. Figure 1 shows a schema of the workflow of milk sample pre-treatment before AR extraction.

Below, we provide an overview of the available studies on AR extraction and determination. It is necessary to point out that in view of the great variety of methods as well as differences in individual workflows and details, it is virtually impossible to create a critical revision leading to the extraction of a certain approach as the best one. Moreover, many techniques, especially the novel ones, have only been reported in one or two papers, and the independent validation of their usability is needed before they can be introduced into everyday practice. Hence, the overview below should not be perceived as a critical revision of the best techniques but rather as a list of options, including novel approaches, which can help interested readers get oriented on the topic and gain knowledge of the novel trends in AR analysis in milk. The readers should then kindly refer to the original papers for a more detailed description of the methods.

In the oldest paper included in our review, the authors used centrifugation to remove lipids, followed by protein precipitation with a solution of 20% trichloroacetic acid in methanol. Subsequently, the column was double-washed with hexane to remove residual fat. The obtained extract was evaporated and dissolved in Na_2_-EDTA and then applied to an SPE column before the determination of fluoroquinolones and sulphonamides. During the optimization of this process, pure acetonitrile, 20% acetic acid, and 10% trichloroacetic acid in methanol were tested for protein precipitation, but the solution of 20% trichloroacetic acid in methanol provided the best yields [16].

Based on the testing of the efficacy of acetonitrile, ethyl acetate, and mixtures of acetonitrile–methanol and acetone–hexane in different ratios, McIlvaine buffer (citric acid, sodium hydrogen phosphate, Na_2_-EDTA) and acetonitrile–methanol (70:30) were used in pre-extraction processing for the determination of quinolones and beta-lactams. For the extraction itself, Ultrasound-Assisted Extraction was chosen. Interestingly, the authors used the lyophilization of the milk samples as the first step to prevent the interference of water present in the milk matrix with extraction agents [3].

Another study tested multiple solvents, depending on the antibiotic group, for the determination of (fluoro)quinolones, tetracyclines, and sulfonamides. For (fluoro)quinolones, the acidification of the milk sample with 0.1% formic acid in acetonitrile turned out to be the best solution; for both sulfonamides and tetracyclines, ethanol with acetic acid (96:4) was chosen (for tetracyclines, EDTA was added as a chelating agent). This study showed the difficulty of finding a universal procedure that would support the simultaneous analysis of multiple analyte groups [14].

The simultaneous detection of tetracyclines, sulfonamides, quinolones, and other antibiotic groups in a large-scale multi-residue analysis of 88 veterinary drugs also used a chelating agent (Na_2_-EDTA) and ultrasound-aided acetonitrile extraction followed by subsequent centrifugation and SPE. During optimization, the effectiveness of various extraction solvents (phosphate buffer, ammonium acetate, 5% trichloroacetic acid, methanol, acetonitrile, and 1% acetic acid in acetonitrile) was evaluated. Non-polar analytes (benzimidazoles and steroidal substances) showed low recoveries when water-based buffers were used, while a mixture of acetonitrile and acetic acid degraded the beta-lactam antibiotics to disrupt the nucleus. Acetonitrile turned out to be the best compromise [20].

Other authors extracted fluoroquinolones in milk using a solution of trichloroacetic acid and methanol (2:8), which was followed by mechanical treatment (vortexing, centrifugation), the evaporation of the supernatant, dissolution in water, and pH adjustment. Next, the samples were purified for analysis using magnetic graphene-dispersive solid-phase extraction (MG-DSPE) [11].

Aminoglycosides were extracted from milk samples using a solution of potassium dihydrogen phosphate, Na_2_-EDTA, and 2% trichloroacetic acid. After vortexing and centrifugation, the pH was adjusted to 7.0 using ammonium hydroxide before subsequent SPE [6].

Ammonium acetate was used for the determination of tetracyclines; after centrifugation, the authors also adjusted the pH to 7.0 with acetic acid and ammonium hydroxide before magnetic SPE (MSPE) [18].

Acetonitrile was chosen for the first step of milk sample treatment, which proved to be the most suitable for the determination of macrolide antibiotics by the QuEChERS method (Quick, Easy, Cheap, Effective, Rugged, Safe). Methanol, 0.1% formic acid in acetonitrile, and mixtures of ammonium formate–acetonitrile and 0.1% formic acid–ammonium formate–acetonitrile were also tested [19].

For the pre-treatment and extraction of aminoglycosides and polypeptide antibiotics in milk samples, a 0.25% aqueous solution of trichloroacetic acid was used. After sonication and centrifugation, the pH was adjusted to 6.5 before application to the SPE sorbent [59].

Other authors analyzed polypeptide antibiotics in infant formula milk powder. After dissolving the milk in water, they acidified the sample with formic acid in methanol. Deproteinization was ensured by the use of acetonitrile; lipids were removed by double extraction into hexane. After that, sample preparation was completed using SPE [4].

A mixture of McIlvaine buffer and the chelating agent EDTA, trichloroacetic acid, and hydrochloric acid were used for determining three groups of antibiotics (tetracyclines, fluoroquinolones, and sulfonamides). The supernatant after centrifugation was then purified using rotating-disk sorptive extraction [7].

Another study used a very simple method of milk sample preparation consisting of the precipitation of milk proteins using acetonitrile, centrifugation, and supernatant evaporation, which was followed by dissolving the sample in the mobile phase and filtering it into a vial. This method was used for the analysis of fluoroquinolones, tetracyclines, and their epimers, which are often found in food and the environment as the main degradation products [5].

Other authors used, similar to studies analyzing tetracyclines, chelating agents in their pre-treatment and extraction stages. To disrupt the chelated complexes of the analyte with calcium, they used citrate–phosphate buffer, citric acid, Na_4_-EDTA, and acetonitrile. After centrifugation, the supernatant was subjected to microextraction using deep eutectic solvents [8].

Another study analyzed tetracyclines, beta-lactams, chloramphenicol, and fluoroquinolones using a precipitation reagent of 20% zinc sulfate and 5% sodium chloride to increase the ionic strength of the solution. After the sonication and centrifugation of the solution, the supernatant was subjected to microextraction using magnetic ionic liquids (MILs). During the optimization of the sample preparation, the appropriate volume (volume 0.50–1.50 mL, optimum 1.25 mL) and concentration of the precipitating agent (10–25%, optimum 20%) were investigated, along with the sonication time (1–5 min, optimum 3 min) and the required amount of NaCl (0; 2.5; 5; 7.5, and 10%, optimum 5%) [55].

For the analysis of 132 veterinary drugs from the sulfonamides, beta-lactams, tetracyclines, quinolones, and other groups of antibiotics, milk samples were treated with acetonitrile, McIlvaine buffer, and hexane for lipid removal. This was followed by MSPE centrifugation. The authors highlight the importance of pH adjustment during the sample preparation stage [15].

Milk proteins were precipitated with trichloroacetic acid to determine gentamicin and streptomycin (aminoglycosides). The optimum acid amount was 300 mg per 15 mL of milk. The obtained supernatant was adjusted to pH 10 (showing the best results from the pH range of 4–10) and purified using DSPE [58].

In multi-residual analysis, acetonitrile, centrifugation, supernatant evaporation and its reconstitution in ultraclean water, pH correction to 6.8 using Britton–Robinson buffer, and MSPE were used [56].

Sulfonamides were extracted using ethyl acetate. After centrifugation, the supernatant was evaporated, dissolved in water, and further subjected to ultrasonic-assisted dispersive micro-solid-phase extraction [17].

For sulfonamide determination, lipids were removed using centrifugation in the first step, after which proteins were precipitated with acetonitrile. After centrifugation, the supernatant was processed using magnetic dispersive miniaturized solid-phase extraction [64]. Similarly, for the determination of lincomycin, the supernatant was obtained from milk samples after the addition of acetonitrile and subsequent centrifugation, and SPE was applied using a Cu-based metal–organic framework adsorbent [65].

For the determination of quinolones, milk samples were mixed with trichloroacetic acid. After the centrifugation of the precipitated proteins, the supernatant was filtered through a membrane filter, supplemented with ultrapure water to 50 mL, and prepared for analysis at 4 °C using thermo-sensitive magnetic ionic liquids [66].

Milk was mixed with a 2% zinc acetate solution to precipitate proteins. After centrifugation, the pH of the upper liquid phase was adjusted to 10.0 using 0.1 M NaOH and 0.1 M HCl. The sample volume was adjusted to 10.0 mL, and the sample was filtered into an extraction vial and analyzed for trimethoprim [67].

Another method of protein precipitation was used for the determination of amoxicillin. Milk was vortexed with 0.7 M trichloroacetic acid. After centrifugation, the supernatant was filtered through paper filter, which was followed by a salting-out-assisted liquid–liquid extraction [68].

Milk was vortexed with a zinc sulfate solution (30%, *w*/*v*). After centrifugation, the upper liquid phase was collected for subsequent dispersive solid-phase extraction based on covalent organic frameworks (COFs) and analyzed for sulfonamides [69].

Sulfonamides were analyzed also in another study in which the authors precipitated milk proteins using K_4_Fe(CN)_6_·3H_2_O and ZnSO_4_·7H_2_O. After thorough mixing and centrifugation, the supernatant was diluted with water to 30 mL, and the pH was adjusted to 4.0 before employing magnetic SPE [70].

Another study analyzed tetracyclines. Milk was deproteinized with 10% acetic acid. After centrifugation, the supernatant was subjected to magnetic solid-phase extraction using *Azadirachta indica* for the green synthesis of magnetic nanocomposites, providing greater adsorption capacity towards tetracyclines [71].

Tetracyclines were analyzed also by other authors, who vortexed milk samples with acetonitrile, centrifuged the mixture, and evaporated the resulting supernatant under a gentle nitrogen stream until a target sample volume of 0.5 mL was achieved. Then, dispersive SPE was applied for tetracycline determination using a biosorbent [72].

Chloramphenicol was analyzed in milk, which was vortexed with 0.5% ascorbic acid and methanol. After centrifugation, the supernatant was subjected to fabric-phase sorptive extraction [73].

Tetracycline, oxytetracycline, and ciprofloxacin were determined simultaneously. Proteins were precipitated with 20% trichloroacetic acid in acetonitrile and methanol to precipitate the proteins. After cooling for 10 min in a refrigerator and subsequent centrifugation, the supernatant was filtered through a 0.20 μm PTFE syringe filter, the volume of the solution adjusted with methanol to 6 mL, and it was stored till the final analysis [74].

**Table 2:** The list of studies analyzing antimicrobials in milk (in the order of the number of different antibiotic groups per analysis).

Antibiotic Group	Analytical Method	Column Parameters	Mobile Phase	Recovery [%]	Precision [%]	R/R^2^	LOQ	Ref.
Amphenicols Beta-lactams (Fluoro)quinolones Lincosamides Macrocyclic lactones Macrolides Pleuromutilines Quinoxalines Sulfonamides Tetracyclines Trimethoprim	HPLC-MS/MS	Acclaim 120 C18 (100 × 2.1 mm; 3 µm)	0.5% FA AcN MeOH	82–120	˂20	0.990–0.999	0.05–1 µg/kg	[15]
Amphenicols Beta-lactams (Fluoro)quinolones Lincosamides Macrolides Sulfonamides	UPLC-MS/MS	Acquity UPLC HSS T3 (100 × 2.1 mm; 1.7 µm)	0.1% FA AcN	37.1–118.0	2.3–19.2	0.9902–0.9998	0.1–50 µg/kg	[10]
Beta-lactams (Fluoro)quinolones Ionophores Nitroimidazoles Sulfonamides Tetracyclines	LC-HRMS (Orbitrap) LC-HRMS (TOF)	Kinetex C18 (150 × 2.1 mm; 2.6 µm)	FA H_2_O AcN	3.4–771.1	0.3–104.8	0.8027–0.9997	Only LOD 0.5–50 µg/kg	[60]
Beta-lactams (Fluoro)quinolones Macrolides Sulfonamides Tetracyclines	UPLC-MS/MS	C18 (100 × 2.1 mm; 1.7 µm)	AmF 0.1% FA AcN MeOH	85.91–106.95	1.16–9.2	0.9958–0.9992	0.13–0.64 µg/kg	[56]
Beta-lactams (Fluoro)quinolones Macrolides Sulfonamides Tetracyclines	LC-MS/MS	CAPCELL PAK C18 MG III (150 × 2.1 mm; 5 µm)	0.1% FA AcN	63.1–117.4	3.3–17.6	>0.9930	0.5–10 µg/kg	[20]
Amphenicols Beta-lactams (Fluoro)quinolones Tetracyclines	HPLC-DAD	Zorbax-SB-Aq C18 (100 × 4.6 mm; 3.5 µm)	0.2% FA MeOH	79–91	3.6–5.2	0.994–0.999	0.29–0.71 µg/kg	[55]
(Fluoro)quinolones Sulfonamides Tetracyclines	HPLC-DAD	RP-C18 (250 × 4.6 mm; 5 µm)	0.2% TCA MeOH AcN	85.5–106.4	3.7–9.9	0.990–0.998	9.2–46.6 µg/kg	[7]
	UPLC-TOF/MS	Brownlee HRes DB PiPh (50 × 2.1 mm; 1.9 µm)	1% FA AcN	88.5–114.1		0.990–0.999	1.9–76.0 µg/kg	
(Fluoro)quinolones Sulfonamides Tetracyclines	LC-MS	SYMMETRY C18 (75 × 4.6 mm; 3.5 µm)	0.1% FA AcN	62–108	3.8–15.0	0.9625–0.9968	2.5–25 µg/kg	[14]
Aminoglycosides Polypeptides	LC-Q-Orbitrap	Poroshell 120 HILIC (100 × 2.1 mm; 2.7 µm)	1% FA AmF AcN	82–96	4.9–7.5	>0.99	10–33 µg/kg	[59]
Beta-lactams (Fluoro)quinolones	UPLC-MS/MS	Acquity UPLC BEH C18 (50 × 2.1 mm; 1.7 µm)	0.1% FA AcN	91–99	1.7–12.3	>0.96	1.5–8.7 µg/kg	[9]
Beta-lactams (Fluoro)quinolones	UHPLC-MS/MS	Acquity UPLC BEH TM C18 (100 × 2.1 mm; 1.7 µm)	AmF MeOH	96.0–104.5	0.3–7.1	0.979–0.999	0.3–2.0 µg/kg	[3]
(Fluoro)quinolones Sulfonamides	UPLC-MS/MS	Acquity UPLC BEH C18 (50 × 2.1 mm; 1.7 µm)	0.1% FA MeOH	61–115	6.3–10.7	0.9923–0.9980	0.01–1.68 µg/kg	[16]
(Fluoro)quinolones Tetracyclines	HPLC-PDA	Shim-pack GIS C18 (250 × 4.6 mm; 5 µm)	0.05 M OxA AcN MeOH	97.35–107.80	0.25–3.22	0.9995–0.9999	0.09–0.20 µg/kg	[74]
Sulfonamides Tetracyclines	UHPLC-MS/MS	SB-C18 (50 × 2.1 mm; 1.8 µm)	0.1% FA AcN	73.3–87.78	1.17–7.39	0.9991–0.9999	2.24–10.43 µg/kg	[5]
Amphenicols	LC-MS/MS	LMA-MAA-EDMA (15 cm × 250 µm i.d.)	H_2_O MeOH AcN	95.8–100.2	0.39–2.20	0.994–0.995	Only LOD 0.02–0.045 µg/kg	[12]
Amphenicols	HPLC-DAD	Luna Omega C18 (250 × 4.6 mm; 5 µm)	0,1% TFA MeOH AcN	93–106	˂4.1	0.9942	25.0 µg/kg	[73]
Aminoglycosides	HPLC-MS/MS	Zorbax SB Aq C18 (100 × 4.6 mm; 3 µm)	0.2% FA AmAc	87–93	4–6.1	0.998–0.999	0.18–0.61 µg/kg	[58]
Aminoglycosides	LC-MS/MS	ZIC-HILIC (50 × 2.1 mm; 3.5 µm)	AmF FA MeOH	73.4–94.1	5–10	0.9950–0.9995	7–90 µg/kg	[6]
Beta-lactams	HPLC-UV; MS	Promosil C18 (150 × 4.6 mm; 5 µm)	AcN MPoPh DAmPh	77.4–90.3	3.1–7.4	>0.999	0.13 µg/kg, 0.17 µg/kg	[13]
Beta-lactams	HPLC-CAD	Hypersil Gold C18 (250 × 4.6 mm; 5 µm)	0.1% FA AcN	98.9 ± 2.1	1.9–2.2	0.994	284.4 µg/kg	[68]
	LC-MS/MS	Kinetex Phenyl-Hexyl (50 × 2.1 mm; 1.7 µm)	0.1% FA MeOH	99.7 ± 0.4	1.6–3.0	0.999	3.5 µg/kg	
(Fluoro)quinolones	HPLC-DAD	C18 (150 × 4.6 mm; 5 µm)	AcN 0.05% PhA TEA	86.3–106.3	5.4–9.4	0.9993–0.9998	0.2–0.8 ng/g	[11]
(Fluoro)quinolones	HPLC-UV-Vis	XDB C18 (150 × 4.6 mm; 5 µm)	MeOH AcN 0.5% PhA	95.33–100.18	0.79–4.95	0.9991–0.9998	0.087–0.193 µg/kg	[66]
Lincosamides	HPLC-MS/MS	Acquity UPLC BEH C18 (50 × 2.1 mm; 1.7 µm)	MeOH 1% FA	92.3–97.2	0.25–1.96	≥0.99	Only LOD 0.013 µg/kg	[65]
Macrolides	HPLC-MS/MS	ZORBAX Eclipse plus C18 (150 × 2.1 mm; 3.5 µm)	0.1% FA AmF AcN	62.27–115.28	3.18–7.72	0.9987–0.9999	1.1–4.0 µg/kg	[19]
Polypeptides	HPLC-MS/MS	Poroshell 120 SB C18 (150 × 4.6 mm; 2.7 µm)	0.1% FA MeOH	82.8–101.2	2.2–8.8	0.9958–0.9994	20–50 µg/kg	[4]
Sulfonamides	LC-HRMS	Acquity UPLC HSS T3 (100 × 2.1 mm; 1.8 µm) Thermo AQ C18 (150 × 2.1 mm; 1.7 µm)	0.1% FA MeOH	68.8–112.6	1.08–8.0	0.9979–1.0000	0.01–0.5 µg/kg	[57]
Sulfonamides	HPLC-DAD	Thermo AQ C18 (150 × 4.6 mm; 3 µm)	FA MeOH AcN	76.7–100.0	1.0–6.3	0.9982–0.9995	3.11–4.02 µg/kg	[17]
Sulfonamides	HPLC-DAD	ZORBAX C18 (100 × 4.6 mm; 5 µm)	1% AcA AcN	62–71	3.2–7.0	0.99	0.37–0.63 µg/kg	[69]
Sulfonamides	HPLC-DAD	C18 (150 × 4.6 mm; 5 µm)	0.02% AcA AcN	87.0–98.5	0.9–5.2	>0.995	Only LOD 0.03–0.06 µg/kg	[64]
Tetracyclines	HPLC-MS/MS	Luna C18 (150 × 4.6 mm; 5 µm)	0.1% FA MeOH	47–80	Not available	Not available	0.015–0.15 µg/kg	[8]
Tetracyclines	HPLC-MS/MS	ZORBAX Eclipse Plus C18 (100 × 3.0 mm; 1.8 µm)	1% AcA AcN	87.0–101.8	1.1–5.4	0.9965–0.9989	0.017–0.183 µg/kg	[18]
Tetracyclines	HPLC-UV	Welchrom C18 (250 × 4.6 mm; 5 µm)	80% OxA MeOH for OTC	94–96	3–6	0.998–0.999	0.6–1.5 µg/kg	[71]
			39% MeOH 1% PhA for CTC					
Tetracyclines	HPLC-UV	Purosher Star RP-18 (150 × 4.6 mm; 5 µm)	0.2% TFA AcN	83.1–109.9	7.02–13.32	0.9994–0.9997	15.0 µg/kg	[72]
Trimethoprim	HPLC-UV	ZORBAX Eclipse Plus (100 × 4.6 mm; 3.5 µm)	0.03% FA AcN MeOH	81.6–98.0	3.5–5.7	0.994	10.0–25.0 µg/kg	[67]

AcA, acetic acid; AcN, acetonitrile; AmAc, ammonium acetate; AmF, ammonium formate; CAD, Charged Aerosol Detector; CTC, chlortetracycline; DAD, diode array detection; DAmPh, diammonium phosphate; FA, formic acid; HPLC, high-performance liquid chromatography; HRMS, high-resolution mass spectrometry; LC, liquid chromatography; LMA-MAA-EDMA, poly-lauryl methacrylate-co-methacrylic acid-co-ethylene glycol dimethacrylate; LOD, limit of detection; LOQ, limit of quantification; MeOH, methanol; MS, mass spectrometry; MPoPh, monopotassium phosphate; MS/MS, tandem mass spectrometry; OxA, oxalic acid; OTC, oxytetracycline; PDA, Photo Diode Array Detector; PhA, phosphoric acid; R/R^2^, Correlation Coefficient; TEA, triethylamine; TOF, Time of Flight; TFA, Trifluoroacetic Acid; UHPLC, ultra-high-performance liquid chromatography; UPLC, ultra-performance liquid chromatography; UV, UltraViolet.

#### 4.1.2. Extraction of Antibiotic Residues

In this stage of sample preparation, the actual extraction of ARs into an HPLC-compatible solvent is performed. These procedures aim to obtain a final eluate that is subsequently evaporated, reconstituted, most commonly in the mobile phase, and filtered into a vial. In this way, the sample is prepared for analysis by liquid chromatography. Solid-phase extraction on various sorbents plays a pivotal role in most studies, although some have also used liquid–liquid extraction. For years now, there has been a tendency towards the miniaturization of routine methods and a reduction in the associated costs, which also leads to the lower environmental burden of these analyses, as lesser amounts of organic solvents are used. For this reason, modern methods of sample preparation using microextraction, online techniques, magnetic nanoparticles, or ionic liquids are on the rise.

For the solid-phase extraction of a smaller number of different ARs, some authors chose more versatile HLB cartridges with balanced hydrophilic and lipophilic properties (hydrophilic–lipophilic-balanced). This type of cartridge is suitable for most analytes (acidic, basic, and neutral) as it can be used across the entire pH range (0–14) [4,10,16,75]. In large-scale multi-residue analyses determining tens to hundreds of different substances, SPE runs into a multitude of problems. This is due to a large number of analytes of different physicochemical characteristics, interactions between hydrophobic and hydrophilic components, and issues associated with the elution process (i.e., with the affinity of the substance for the sorbent being higher than that for the elution solvent) [15]. Hence, ion-exchange sorbents are often used for SPE, the retention mechanism of which is based on the electrostatic interaction of the polar groups of the analyte and the sorbent. One can choose WCX (Weak Cation-eXchanger) containing weak cation-exchange groups for the sorption of basic substances (this method is applicable, e.g., to the determination of aminoglycosides and polypeptide antibiotics [59]) or SCX (Strong Cation-eXchanger) containing strong cation-exchange groups (applicable, e.g., to the determination of aminoglycosides antibiotics) [6]. However, both types of SPE cartridges present some disadvantages. SCX does not provide quantitative elution due to the strong binding of aminoglycoside antibiotics. WCX, on the other hand, requires the careful adjustment of the pH of the sample being analyzed, and differences in the interaction of different aminoglycoside antibiotics with ion-exchange groups have also been observed [6]. The use of MCX (Mixed Cation-eXchanger) or MAX (Mixed Anion-eXchanger) represents additional options. These sorbents provide two ways of retention, i.e., ion exchange as well as reverse phase, thus being capable of the adsorption of a wide range of chemicals. For extensive multi-residual analyses, however, this approach may fail to be sufficiently quantitative [10]. PCX (Polymeric Cation-eXchanger) is characterized by a greater surface area and can be used for the extraction of sulfonamides as weakly alkalic compounds [57]. Possible extraction solvents include water, methanol, their mixtures, or various concentrations of formic acid in water, methanol, or their mixtures [4].

Online SPE represents a time-saving and cost-saving alternative to the classic SPE. This automated method (automated turbulent flow cyclone chromatography sample clean-up system) is fast, efficient, sensitive, and environment-friendly. Moreover, it largely eliminates the human factor. As a part of the online SPE optimization for testing a wide range of analytes (eighty-eight antimicrobials and their metabolites—e.g., twenty sulfonamides, seven macrolides, fifteen quinolones, eight penicillins, thirteen benzimidazoles, four tetracyclines), the authors tested the suitability of two types of turboflow SPE columns (polymer-based column and silica-bonded phases), finding that the polymer-based SPE column performed better in their setting. Other optimized parameters included the sample injection volume into the SPE column (100 µL optimum), the solvent used in the sample clean-up stage (water performed best), the flow rate (0.3 mL/min), and last but not least, the elution solvent (acetonitrile–water 60:40) [20].

Rotating-disk sorptive extraction using a styrene–divinylbenzene sorbent is another possible modification of the SPE method, showing the best performance across a wide range of sorbents (silica-octyl, silica-octadecyl, silica-cyanopropyl, Florisil, Oasis HLB, silica). The authors determined oxytetracycline and its 4-epimer, enrofloxacin, ciprofloxacin, sulfadoxine, and trimethoprim in cow’s milk [7].

Another frequently used method, QuEChERS, brings cost, time, and solvent savings. The method is based on the principle of dispersive solid-phase extraction (d-SPE), where salts are added to the matrix to remove water and other unwanted matrix components, such as proteins or lipids. QuEChERS was originally developed for foods of a simpler composition; however, the method was further optimized and the workflow modified to enable the analysis of complex foods [3,9,12]. Typically, the method consists of the extraction stage and a clean-up stage (also partitioning, salting-out stage). Methanol, acetonitrile, McIlvaine buffer, or ethyl acetate is often chosen as an extraction solvent [10,19,60]. In the clean-up stage, sodium sulfate, magnesium sulfate, primary secondary amine (PSA), sodium acetate, or PestiCarb is often used [3,9,19], similarly, Flori silica or graphitized carbon black [10] and silica-octyl or Z-Sep+ and their combination [12]. Each of these sorbents, however, may show advantages and disadvantages for a particular analysis (such as insufficient separation of lipid and carbohydrate components in the case of Flori silica or insufficient purification from lipophilic substances and excessive adsorption of acidic analytes when using PSA). Despite this, PSA is a suitable sorbent for removing fatty acids and sterols from the matrix. The application of magnesium, sodium or ammonium sulfate, sodium chloride, EDTA, ammonium formate, acetate, and ammonium chloride in the salting-out step is also reported [9,60]. The selection of suitable salts is important for obtaining correct results. Various hydrogen phosphates (K_2_HPO_4_, KH_2_PO_4_, Na_2_HPO_4_∙12H_2_O, NaH_2_PO_4_∙H_2_O, (NH_4_)_2_HPO_4_, (NH_4_)H_2_PO_4_) can be also used. The QuEChERS method was used for the determination of 201 veterinary drugs, which is obviously a difficult task considering the extremely high or low polarity of individual analytes, low pH stability, poor detection sensitivity, and chemical bonds between the analyte and other matrix components playing a role. Tetracyclines, penicillins, and cephalosporins showed the best recoveries of the analyzed substances [60].

More recent methods include the use of molecularly imprinted polymers (MIPs) in the SPE step. MIPs have been shown to exhibit greater specificity and selectivity than conventionally used SPE sorbents, thereby achieving higher recoveries. In-house MIPs can be used for matrix solid-phase dispersion extraction [13]; commercially produced SPE columns with MIPs are, however, also available [6].

Selective surface molecularly imprinted polymer matrix solid-phase dispersion was employed for the analysis of beta-lactam antibiotics. Of the wide range of extraction solvents tested, dichloromethane performed best as the washing solvent, and the mixture of methanol–10% acetic acid was the most effective elution solvent [13].

The use of magnetic nanoparticles [11,15,18] or magnetic ionic liquids (MILs) [55,56] is also on the rise. For example, magnetic graphene in combination with d-SPE as a special sorption material has been used. Graphene is a nanomaterial with a large surface area exhibiting high stability, absorption capacity, hydrophobicity, and resistance over a wide pH range. Separation on modified graphene particles is based on electrostatic forces that change with pH. This method was originally used for the determination of pesticides, organic contaminants, and metals [11]. Maintaining a suitable pH is important for optimizing the adsorption and desorption efficiency. The principle of the method lies in the binding of the analyte to the magnetic adsorbent, which is followed by the separation of the magnetic particles using an externally applied magnetic field and the subsequent release of the monitored substances into a new solvent, which is used for HPLC analysis [18]. The synthesis of Fe_3_O_4_ nanoparticles and their combination with polystyrene to form magnetic hypercrosslinked polystyrene HCP/Fe_3_O_4_ was applied to a multi-residual analysis of 132 veterinary drugs [15].

Air-Assisted Liquid–Liquid MicroExtraction (AALLME) in combination with new-generation ionic liquids was employed for the determination of seven multiclass antibiotics. The previously developed AALLME method helped to address the drawbacks of conventional LLE methods; the use of ionic liquids, representing so-called green solvents and their utilization, sought to reduce the consumption of routinely used toxic solvents. Ionic liquids effectively bind particles of polymeric materials. Imidazolium, pyridinium, and ammonium-based ionic liquids are the most commonly used. The authors improved this method by using magnetic nanoparticles, namely 1-hexyl-3-methylimidazolium tetrachloroferrate [C_6_MIM][FeCl_4_] as an extraction agent. Subsequently, they used an external magnet for the extraction of the magnetic particle–analyte complex, and subsequently, they released the analytes by reverse extraction into a methanol–acetonitrile–0.2% formic acid solution. This method was found to be the best of the tested approaches—they tested three MILs ([C_4_MIM][FeC_l4_], [C_6_MIM][FeC_l4_], [C_4_MIM][FeCl_2_Br_2_]), multiple volumes of the extraction solvent (70, 80, 90, 100, and 110 µL; optimum 100 µL), the number of extraction repetitions (1–6×, 4× optimum); and the suitable pH (tested range 4–9, optimum 6.3–6.9) [55].

The use of ionic liquids in conjunction with Dispersive Liquid–Liquid Microextraction (DLLME) with polyvinylpyrrolidone as a sorbent for the determination of aminoglycosides was evaluated in another study. The authors found 75 mg of polyvinylpyrrolidone (tested range 25–150 mg) and 20% NaCl addition to be the optimum amounts for this purpose. The addition of salt reduces the solubility of the analytes in the aqueous phase, may reduce the solubility of the organic solvent in the aqueous phase, and enhances the adsorption of the analytes to the sorbent. The method turned out to be unusable with NaCl concentrations below 10%, but the recoveries kept increasing with a salt content up to 20%, after which they remained stable. Methanol was identified as the optimum elution solvent (acetone, acetonitrile, and methanol were tested), and the optimum elution volume was 1.25 mL (tested range of 0.5–1.5 mL). [(C_6_)_3_C_14_P][Cl] proved to be a better extraction solvent than [(C_6_)_3_C_14_P][Br], with the optimum extraction volume being 85 µL (tested range of 85–150 µL) [58].

Imidazole-based ionic liquid-modified magnetic chitosan nanoparticles were applied in a multi-residual analysis of 22 veterinary drugs. Chitosan is a low-cost substance with a high adsorption capacity for both organic and inorganic components of various matrices. It is also highly sustainable—it is classified as a biodegradable and non-toxic biopolymer. However, its poor mechanical resistance and lower stability in acidic conditions appear to be disadvantages. It was used to create a novel nanomaterial, Fe_3_O_4_@CS-IL NPs, by combining it with Fe_3_O_4_. This nanomaterial was subsequently used for magnetic SPE [56].

Chitosan-based polymeric ionic liquids were also combined with ultrasonic-assisted dispersive micro-solid-phase extraction. The authors found the optimum sample pH to be 5–6 (from a tested range of 4–9), the optimum sorbent amount to be 20 mg (from a range of 5–30 mg), the best extraction time to be 15 min (tested range 5–30 min), and the most suitable extraction temperature to be 35 °C (tested range 25–50 °C). The most effective elution was achieved using 10 min (tested range 2–20 min) sonication with 1 mL of 5% ammonia in methanol and water (1:1) [17].

Covalent organic framework (COF) nanoparticles were prepared through an inexpensive and fast process and employed as an efficient sorbent in dispersive SPE (DSPE) of sulfonamide antibiotics from raw milk samples. This step was followed by MIL-based DLLME, and the extracted analytes were determined using HPLC-DAD. This combined method can provide the effective extraction of sulfonamides from a complex sample such as raw milk due to the porous structure of COF. The use of MILs in the DLLME step eliminates the need for centrifugation and makes the method fast and green (due to their low toxicity in comparison with conventional halogenated solvents). Furthermore, the use of MILs eliminates the need for the vaporization of the extraction solvent before injection into the HPLC system [69].

Polypropylene glycol-based thermo-sensitive MILs, together with an aqueous biphasic system (ABS), were used to construct novel thermo-sensitive MILABSs for the highly sensitive determination of trace amounts of quinolone antibiotics in milk samples. The ABS method is time-saving, energy-saving, low-cost, and environment-friendly, without the application of any organic solvents. The efficient phase separations were simply achieved by applying an external magnetic field, the whole analytical method was completely free of any organic solvent, and the thermo-sensitive MILs could also be reused for the next analytical process [66].

In another study, a novel approach was used—the authors investigated the effect of a deep eutectic solvent based on terpenoids and carboxylic acids (hexanoic, octanoic, nonanoic, oleic, tetradecanoic, octadecanoic), followed by re-extraction into the water phase. This method is classified as a microextraction technique, aiming to miniaturize the methods, make them faster, improve selectivity, and maintain sustainability within laboratory practice. Microextraction was carried out using the terpenoids menthol, thymol, and vanillin. The authors reported thymol and vanillin to show superior results to menthol due to the presence of an aromatic ring in their molecule. Due to the lower melting point, thymol was chosen for the analysis [8].

A sol–gel poly(ethylene glycol)-poly(propylene glycol)-poly(ethylene glycol)-modified cellulose fabric-phase sorptive extraction (FPSE) membrane was exploited for the analysis of chloramphenicol in milk samples prior to high-performance liquid chromatography coupled with diode array detection (HPLC-DAD) analysis. The adsorbent demonstrated good material performance, indicating that it could be exploited as a potential material in the development of the FPSE technique with high analytical performance. It has been effectively applied to the determination of selected drugs in milk samples [73].

An alginate hydrogel bead was developed to extract two sulfonamides from milk samples. The novel magnetic dispersive miniaturized solid-phase extraction (M-D-μSPE) adsorbent consisted of ZnO and an MIP coated with functionalized magnetite nanoparticles embedded in alginate (ZnO/Fe_3_O_4_@SiO_2_-NH_2_/MIP/alginate). The extracted target analytes were determined by HPLC-DAD [64].

A green magnetic Ag/GO-Fe_3_O_4_ nanocomposite was synthesized using *Azadirachta indica* leaf extract and used for the residual extraction of chlortetracycline and oxytetracycline from milk samples. This “green” nanocomposite provides more adsorption capacity toward tetracyclines due to its increased porosity, surface-to-volume ratio, and interaction sites. After the magnetic SPE of tetracyclines, quantification was carried out by HPLC-UV [71].

Research on environment-friendly sorbents led to the use of the mucilage of hairy basil seeds (*Ocimum basilicum* L.) for the extraction and preconcentration of tetracyclines in cow’s milk samples. In this experiment, the authors used unmodified hairy basil seeds as a green biosorbent for DSPE before the quantitative analysis of tetracyclines by HPLC-UV. An ultrasound-assisted DSPE was used to increase the extraction efficiency. This method was powerful, simple, low-cost, versatile, environmentally friendly, and reusable [72].

### 4.2. Determination of ARs by Liquid Chromatography

The analytical method is chosen on the basis of the physicochemical properties of the matrix and analyte under investigation. The correct choice of a stationary phase column, allowing for the most efficient separation of the components of the analyzed sample, is a key factor. So is the choice of the mobile phase, which depends on whether the analysis is performed on a normal or reverse-phase column. Most current operating procedures, scientific papers, and application notes use reverse-phase columns and polar solvents [76,77,78]. Table 2 summarizes the parameters of methods used for AR analysis, with the papers being sorted top to bottom according to the number of analyzed groups of antibiotics.

#### 4.2.1. Stationary Phase

Most authors use C_18_ stationary phase columns due to their excellent separation efficiency, sensitivity, resolution, and peak tailing suppression. One of the most widely used columns for this purpose, ZORBAX Eclipse Plus C_18_, is a reverse phase containing highly porous particles [18,19]. Other frequently used columns include the Acquity UPLC BEH C_18_ containing a hybrid stationary phase with ethylene bridges linking silanol groups [3,9,16], ZORBAX SB Aq C_18_ based on the StableBond technology allowing the binding of alkyl phases [55,58], Poroshell 120 SB C_18_ containing a solid core with a porous surface structure [4], Acquity UPLC HSS T3 with a “High-Strength Silica” character of the particles based on 100% silica gel and a T3 technology of C_18_ chain binding enabling the retention of polar compounds [10,57], CAPCELL PAK C_18_ MG exhibiting the enhanced polarity of the stationary phase surface, which ensures the retention of highly polar substances [20], SYMMETRY C_18_ with monofunctionally bound C_18_ ligands on high-purity base-deactivated silica [14], the polar-encapsulated Thermo AQ C_18_ column based on solid-core technology [17,57], and other columns based on the Luna C_18_ [8], Acclaim 120 C_18_ [15], Brownlee HRes DB BiPh [7], or Kinetex C_18_ [60] stationary phases. In some works, other column types were used, such as HILIC working on the principle of hydrophilic ionic stationary phase interactions or a special monolithic column prepared from LMA-MAA-EDMA (poly-lauryl methacrylate-co-methacrylic acid-co-ethylene glycol dimethacrylate), which was used in capillary liquid chromatography [12]. Detailed characteristics of the columns used in the aforementioned studies, including the mobile phases and the antibiotic groups analyzed, are presented in Table 2.

#### 4.2.2. Mobile Phase

Nowadays, most separations are performed on reverse-phase columns with polar mobile phases. Methanol or acetonitrile are often used as organic phases [16,19,20]. Considering that mass spectrometry (MS) is nowadays the routinely used method, especially in positive electrospray ionization mode (ESI+), both water and organic phases are usually enriched with organic acids (most commonly, formic or acetic acid) at concentrations of 0.1–1%, their salts (ammonium formate or ammonium acetate), or a combination of both. These modifiers are mainly needed to improve the peak shape and resolution (suppression of tailing), ionization, and sensitivity during mass spectrometry detection [4,6,16,18,19,20,57]. Some authors prefer methanol over acetonitrile due to improved separation [6,57], while others find acetonitrile more useful due to better elution capacity and, therefore, its low consumption [19].

#### 4.2.3. Detection Techniques

As mentioned above, MS is currently the most commonly employed detection technique (see Table 2), most often in the form of a tandem of two mass spectrometers coupled through a collision cell (MS/MS, also TQD = triple quadrupole or QqQ), although Orbitrap MS or Time-of-Flight (TOF) variants of MS can be also used. Other detection techniques (fluorescence, FLD; diode array detection, DAD; electrochemical detection, ECD) suffer from high detection limits and a weaker response to certain analytes [19,59,60].

Electrospray ionization is typically a method used for polar compounds, while in medium or non-polar analytes, atmospheric-pressure chemical ionization or atmospheric-pressure photoionization is usually preferred. For antimicrobials, positive-mode ESI, which uses the addition of acidifying agents as mentioned above, is most common. Negative-mode electrospray ionization (ESI-), in which basic mobile phases (ammonium hydroxide or ammonium acetate) are preferred, is much less common but still has its place, e.g., for the determination of amphenicols [12,19,20].

## 5. Analysis of Real Milk Samples

Some scientific publications on the development of methods for the determination of ARs in milk apply the developed method to the analysis of a (larger or smaller) number of real milk samples to demonstrate the practical applicability of the newly developed method. Table 3 provides estimates of the prevalence of contamination with antibiotic residues in individual countries based on the published papers. We can see that this prevalence ranges from 0% to 100%, i.e., some studies detected ARs in none of the analyzed samples (except for the positive controls, of course), while others found them in all samples. Below, detailed information about the ARs (concentration of individual antibiotics) in individual studies, along with the MRLs reported in those studies, is provided.

Thirteen raw cow’s milk samples from local farmers and supermarkets in China were evaluated. Twelve samples were found to contain sarafloxacin, gatifloxacin, lomefloxacin, enoxacin, sulfadiazine, sulfamerazine, sulfameter, N_4_-acetylsulfadiazine, and/or N_4_-acetylsulfamerazine in amounts of 13–36 µg/kg. Ofloxacin and enrofloxacin were present in amounts of 14–24 µg/kg in eight samples. Ciprofloxacin (14–44 µg/kg) and N_4_-acetylsulfamethoxazole (8–29 µg/kg) were present in six samples. In all samples, the residues were below the MRLs, meeting the limits for inhibitory substances in milk according to EU legislation (see Table 1) [16]. Ciprofloxacin was detected in one out of sixteen milk samples from China; the level (6 µg/kg), however, met the MRLs (100 µg/kg) for fluoroquinolones [11]. In one of the ten tested samples of cow’s milk from Chile, enrofloxacin was detected (8.5 ± 0.5 µg/kg) [7]. The presence of quinolones (enrofloxacin, ciprofloxacin, marbofloxacin) and beta-lactam antibiotics (amoxicillin, ampicillin, penicillin G, dicloxacillin, cephalonium) was evaluated in 28 samples from Spanish cows, some of which were treated with antibiotics. They detected 2–34 µg/kg, which complied with the standards [3]. Ciprofloxacin and enrofloxacin residues below the MRLs were revealed in six samples from more than six hundred analyzed in Brazil. In addition, oxytetracycline and tetracycline were detected in six samples. In one sample, the oxytetracycline content (981 µg/kg) exceeded the MRLs by almost ten times [14]. Fifteen cow’s milk samples (eleven raw, three pasteurized, one UHT) from Bangladesh were analyzed for tetracycline, oxytetracycline, and ciprofloxacin. Tetracycline and/or oxytetracycline were detected in six raw, two pasteurized, and one UHT milk samples, respectively. Similarly, ciprofloxacin was detected in seven raw samples, one pasteurized sample, and one UHT milk sample. After a comparison of all samples with the EU MRLs, excessive levels of tetracycline, oxytetracycline, and ciprofloxacin were detected in six (40%), eight (50%), and four (25%) milk samples, respectively, with some levels exceeding the MRLs by two to four times [74].

One hundred and thirty milk samples from Turkey were analyzed for tetracycline, 4-epitetracycline, 4-epioxytetracycline, 4-epichlorotetracycline, and ciprofloxacin. Seventy-two analyzed samples were positive for at least one of these antibiotics (colostrum—four positives out of ten samples; UHT milk—twenty-four out of thirty-one; raw goat’s milk—thirteen out of twenty-one; and raw cow’s milk—thirty out of sixty-eight). The total tetracycline/4-epitetracycline concentrations ranged between 0.01 and 0.17 µg/kg. In six raw goat’s milk samples (out of twenty-one) and six raw cow’s milk samples (out of sixty-eight), the permitted combined tetracycline and its 4-epimer levels were exceeded [5]. All 10 examined cow’s milk samples coming from Thailand supermarkets were positive for oxytetracycline, and two samples were also positive for tetracycline and chlortetracycline; however, the MRLs were not exceeded in any of these samples [72]. In total, 241 cow’s milk samples (133 raw and 108 pasteurized) from Iran were evaluated for residues of inhibitory substances. Residues were found in 124 samples (73 raw and 51 pasteurized). The samples contained tetracycline, 4-epitetracycline, oxytetracycline, 4-epioxytetracycline, 4-epichlorothracycline, amoxicillin, and/or ciprofloxacin at levels of 0.54–97.18 µg/kg. Some samples (24 raw and 15 pasteurized) exceeded the MRLs for the group total [56]. Out of thirty samples from Iranian supermarkets, six milk samples were positive simultaneously for oxytetracycline (89–149 µg/kg) and tetracycline (56–112 µg/kg). In addition, one sample showed the presence of chloramphenicol at 41 µg/kg [55]. Amphenicols (chloramphenicol, thiamphenicol, and florfenicol) were monitored in six milk samples from Taiwan. The authors detected the presence of these antibiotics in none of the analyzed samples [12].

Thirty-three samples of retail milk from China were analyzed for antibiotics (sulfamethoxypyridazine, sulfamerazine, cloxacillin, norfloxacin, erythromycin, amoxicillin) as well as the hormonal substances progesterone and testosterone. The concentrations of antibiotic residues in their study ranged from 3.1 to 22.9 µg/kg. Two samples were positive for sulfonamides, one sample for erythromycin, two samples for beta-lactam antibiotics, and two samples for norfloxacin [20]. None of 15 analyzed samples from Iran were positive for sulfonamides [69]. Commercial milk (*n* = 18) samples from China were analyzed. The detected values for the sulfonamides sulfadiazine (0.3–9.7 µg/kg) and sulfamerazine (4.9–7.4 µg/kg) in three milk samples were in line with the EU MRLs [57].

Sixty milk samples from different producers in China were tested for sixteen macrolide antibiotics and four metabolites. In three samples, erythromycin (8.38–17.86 µg/kg) and tilmicosin (12.45–28.18 µg/kg) were detected. These values met the EU MRLs set for milk (40 µg/kg erythromycin, 50 µg/kg tilmicosin) [19]. In total, 30 cow’s and sheep’s milk samples from Iranian dairies were analyzed for aminoglycoside antibiotics. Two cow’s milk samples were positive for gentamicin (16.3 ± 0.6 and 112 ± 9.2 µg/kg) and seven for streptomycin (13.6 ± 0.3 to 289.1 ± 22.6 µg/kg) [58]. Out of twenty liquid cow’s milk and ten powdered milk samples from supermarkets in China, lincomycin was detected at 10.2 ± 1.5 µg/kg in one milk sample [10].

## 6. Conclusions

The protection of the dairy chain from exogenous foreign substances, such as antibiotic residues, is an important field that has long been at the forefront of food safety concerns. In this review, we provided a brief background on the issue of ARs in milk and associated legislative requirements (highlighting the importance of harmonizing legislative requirements among countries), after which we specifically focused on the analytical methods for AR determination. Besides providing a general workflow on milk sample preparation and subsequent AR determination, we provided an extensive overview of the techniques currently used for this purpose, including emerging and green techniques (reduction in organic solvent consumption, use of sorbents of natural origin, etc.). However, most of these emerging techniques have not been independently validated so far, and future research is needed to verify the results reported by the principal investigators, which can subsequently lead to the introduction of these methods to everyday analytical practice.

Given the extreme range of the characteristics of antibiotic residues, multi-residual analysis still poses a major challenge for analysts. While most studies still analyze only a few selected ARs, attempts to perform such analyses (with greater or smaller success) have already been made, hopefully paving the way not only for future research in this field but possibly also for application in dairy and regulatory practice. This review offers a good starting point for anyone interested in the analysis of antibiotic residues in milk, offering the latest literature and describing a wide range of methods applicable to this purpose.

## Figures and Tables

**Table 3:** Prevalence of antibiotic residues in the cited studies.

Antibiotic Group	Milk Treatment/Origin	Prevalence (%) (Positives/All Samples)	Country of Sample Origin	References
Amphenicols	Cow’s, marketed	0% (0/6)	Taiwan	[12]
	Cow’s, marketed	3.3% (1/30)	Iran	[55]
Aminoglycosides	Raw cow’s and sheep’s	30% (9/30)	Iran	[58]
Beta-lactams	Raw cow’s	28.6% (8/28)	Spain	[3]
	Cow’s, marketed	6.1% (2/33)	China	[20]
	Cow’s (raw, pasteurized)	51.5% (124/241)	Iran	[56]
Lincosamides	Liquid and powder, marketed	3.3% (1/30)	China	[10]
Macrolides	Cow’s, marketed	5% (3/60)	China	[19]
	Cow’s, marketed	3.0% (1/33)	China	[20]
(Fluoro)quinolones	Raw cow’s	28.6% (8/28)	Spain	[3]
	Raw cow’s, raw goat’s, colostrum, UHT	55.4% (72/130)	Turkey	[5]
	Cow’s, marketed	10% (1/10)	Chile	[7]
	Raw cow’s	6.3% (1/16)	China	[11]
	Raw cow’s	1% (6/604)	Brazil	[14]
	Raw cow’s	92.3% (12/13)	China	[16]
	Cow’s, marketed	6.1% (2/33)	China	[20]
	Cow’s (raw, pasteurized)	51.5% (124/241)	Iran	[56]
	Cow’s (raw, pasteurized, UHT)	60% (9/15)	Bangladesh	[74]
Sulfonamides	Raw cow’s	92.3% (12/13)	China	[16]
	Cow’s, marketed	6.1% (2/33)	China	[20]
	Cow’s, marketed	16.7% (3/18)	China	[57]
	Raw cow’s	0% (0/15)	Iran	[69]
Tetracyclines	Raw cow’s, raw goat’s, colostrum, UHT	55.4% (72/130)	Turkey	[5]
	Raw cow’s	1% (6/604)	Brazil	[14]
	Cow’s, marketed	20% (6/30)	Iran	[55]
	Cow’s (raw, pasteurized)	51.5% (124/241)	Iran	[56]
	Cow’s, marketed	100% (10/10)	Thailand	[72]
	Cow’s (raw, pasteurized, UHT)	60% (9/15)	Bangladesh	[74]
